# Ring the yield: regulation of spike architecture by an E3 ubiquitin ligase in crops

**DOI:** 10.1093/jxb/erad281

**Published:** 2023-09-12

**Authors:** Yusheng Zhao, Zhiyong Liu

**Affiliations:** State Key Laboratory of Plant Cell and Chromosome Engineering, Institute of Genetics and Developmental Biology, The Innovative Academy of Seed Design, Chinese Academy of Sciences, Beijing, China; College of Advanced Agricultural Sciences, University of Chinese Academy of Sciences, Beijing, China; State Key Laboratory of Plant Cell and Chromosome Engineering, Institute of Genetics and Developmental Biology, The Innovative Academy of Seed Design, Chinese Academy of Sciences, Beijing, China; College of Advanced Agricultural Sciences, University of Chinese Academy of Sciences, Beijing, China; Hainan Yazhou Bay Seed Laboratory, Sanya City, Hainan Province, China

**Keywords:** RING finger E3 ubiquitin ligase, spike length, TaAIRP2-1B

## Abstract

This article comments on:

Zhang J, Li C, Li L, Xi Y, Wang J, Mao X, Jing R. 2023. RING finger E3 ubiquitin ligase gene *TaAIRP2-1B* controls spike length in wheat. Journal of Experimental Botany 74, 5014–5025.

**Spike architecture involves many key yield-related agronomic traits in crops. E3 ubiquitin ligases play important roles in many biological processes. Using association analysis, Zhang *et al.* (2023) revealed that the wheat E3 ubiquitin ligase TaAIRP2-1B positively regulates spike length by involving an upstream regulator, TaERF3, and a downstream target, TaHIPP3, providing deepened understanding of regulation of spike architecture and an alternative molecular module for designing future wheat with increased yield potential.**

Common wheat (*Triticum aestivum* L.), a staple food crop throughout the world, provides ~20% of calories and protein consumed by humans. Thus, wheat production is highly critical for food security across the world. However, the dramatic increase in the human population and rapid urbanization have reduced the available arable land; therefore, continuously improving wheat yield is more urgent than ever. A better understanding of spike architecture will be very helpful to achieve the goal of continuous yield improvement.

Wheat yield is a complex trait determined by three yield component traits (YCTs), namely thousand kernel weight (TKW), kernel number per spike (KNS), and spike number (SN) (Cao *et al*., 2020). During the wheat domestication and breeding process, favourable yield-related agronomic traits such as large spikes, increased grain size, increased spike number, and increased grain number per spike were selected by farmers and breeders for higher yield. A wealth of studies have been performed to discover genes and regulatory networks controlling these traits (Cao *et al.*, 2020). With recent advances in wheat genomics, a large number of yield-related loci have been identified. Of note, the major stable genetic loci for YCTs in wheat remains largely elusive, so does the regulatory network underpinning YCTs and other yield-related traits, such as spike length, flowering time, and plant height. Spike development involves a series of events, of which the formation of apical spikelet is critical for determining final spikelet number and spike length (Zhang *et al*., 2022). Actually, spike length profoundly influences grain number per spike, with a positive correlation to plant biomass, harvest index, and yield, but a negative correlation to spikelet density (Zhu *et al*., 2018). Notably, there are also studies showing that longer spikes can increase yield without compromising spikelet density (Che *et al*., 2018; Yang *et al*., 2019). As is well known, grain number per spike and grain weight are two antagonistic factors limiting the yield potential (Quintero *et al*., 2018; Wang *et al*., 2022), which could be to some extent alleviated by a larger spike (Zhang *et al.*, 2022). Thus, improving spike length, as presented in the study by Zhang *et al*. (2023), could provide a new idea for enhancing the wheat yield potential and deserves more attention to achieve continuous yield improvement.

Zhang *et al*. (2023) firstly found that *TaAIRP2s* are universally expressed at seedling and jointing stages, with peak expression at the jointing stage in wheat, which led the authors to speculate that TaAIRP2s might have a function in spike development. To examine this hypothesis, Zhang *et al* (2023) sequenced *TaAIRP2* genes from 32 diversified wheat accessions and identified four single nucleotide polymorphisms (SNPs) in the upstream part of the *TaAIRP2-1B* coding region. Based on the four SNPs, two haplotypes of *TaAIRP2-1B* were defined within the wheat collection. Then, the authors developed the molecular marker TaAIRP2-1B-dCAPS and evaluated the relationship between haplotypes of *TaAIRP2-1B* and yield-related traits in 30 environments over 7 years. The results showed that *Hap*-1B-1 is highly associated with spike length in 22 environments. Further analysis showed that transcript levels of *TaAIRP2-1B* in *Hap*-1B-1 are significantly higher than in *Hap*-1B-2, suggesting that *TaAIRP2-1B* has a positive correlation with longer spikes. Consistently, *Hap*-1B-1 was selected as a favourable allele for higher yield during breeding in China. In other crops, panicle length has been reported to play a critical role in improving panicle architecture and grain yield. In fact, panicle length is correlated with many key agronomic traits, such as grain number per panicle and grain density, by directly influencing the number and length of panicle branches (Zhang *et al*., 2015; Jing *et al*., 2018). Thus, the identification of the favourable haplotype *TaAIRP2-1B* for a larger spike in wheat provides breeders with an alternative route to develop high-yield varieties of wheat.

To understand how *Hap*-1B-1 confers higher expression of *TaAIRP2-1B*, the authors performed promoter analysis and revealed that SNPs disrupt the binding site of the repressive factor TaERF3 in *Hap*-1B-1 but not in *Hap*-1B-2. Indeed, yeast one-hybrid analysis validated that TaERF3 could directly bind to the promoter of *TaAIRP2-1B* and represses its expression in *Hap*-1B-2 but not in *Hap*-1B-1. ERF transcription factors play important roles in plant development across many species, such as flowering, spikelet growth, endosperm development, and grain size (Schmidt *et al*., 2014; Liu *et al*., 2022). Here, Zhang *et al.* (2023) reported that TaERF3 negatively regulates *TaAIRP2-1B* expression by targeting the ERF-binding motif in the promoter of the haplotype *Hap*-1B-2 but not in the haplotype *Hap*-1B-1, indicating that TaERF3 may regulate wheat spike length by directly repressing *TaAIRP2-1B* expression in *Hap*-1B-2. This very interesting finding provides insights into how *TaAIRP2-1B* is possibly regulated in wheat. However, it requires extra genetic support and it will be very interesting to genetically confirm in the future if TaERF3 really regulates spike length. Of note, TaERF3 was reported to positively regulate grain weight by suppressing *TaSDIR1-4A* expression in wheat (Wang *et al*., 2020). Thus, it may be worth pyramiding the TaERF3–TaAIRP2-1B–TaSDIR1-4A molecular module to see if this module can simultaneously improve both the spike length and grain weight.

The ubiquitin–proteasome system regulates the stability of substrate proteins and consists of three enzymes: ubiquitin-activating enzyme E1, ubiquitin-conjugating enzyme E2, and ubiquitin ligase E3. Eukaryotes usually possess one or two E1s, tens of E2s, but hundreds of E3 ligases, which mainly consist of a U-box, HECT (Homology to E6-Associsted Carboxyl-Terminus) or RING-type (Really Interesting New Gene) (Lyzenga and Stone, 2012; Callis, 2014). The role of E3 ligase in regulating the agronomic traits of crops has been emerging in recent years (Varshney and Majee, 2022), with most of the studies focusing on the size of panicles and grains. For example, OsCLG1 (Chang Li Geng 1) increases grain size by promoting the degradation of GS3 (Yang *et al*., 2021). TaSDIR1 (Salt and drought-induced RING finger 1) affects grain size (Wang *et al.*, 2020). Here Zhang *et al* (2023) reported that *TaAIRP2* is involved in the regulation of spike length in wheat and encodes a RING-type ubiquitin ligase, similar to its counterpart *AtAIRP2* in Arabidopsis (Cho *et al*., 2011). Consistent with its potential function in the proteasome, TaAIRP2-1B is localized only in the cytoplasm.

In order to explore the possible mechanism by which TaAIRP2-1B regulates spike length, the authors performed yeast two-hybrid screening and found that TaHIPP3 is a physically interacting partner. Transient co-expression in tobacco leaf showed that physical interaction with TaAIRP2-1B promotes TaHIPP3 degradation, as expected considering its role as an E3 ligase. A previous study showed that HIPP proteins regulate endoplasmic reticulum-associated degradation of CKX proteins and cytokinin responses in Arabidopsis (Guo *et al*., 2021). Interestingly, an earlier study reported that CKX increases silique length and regulates pod development in oilseed rape (Liu *et al*., 2018). Moreover, Zhang *et al.* (2023) showed that TaHIPP3s are highly and specifically expressed in the spike, supporting their role in regulating spike development and a possible working model of promoted degradation of TaHIPP3 by TaAIRP2-1B. The finding of enhanced spike length in *TaAIRP2-1B*-overexpressing rice lines highlights a conserved function of TaAIRP2 in regulation of spike length. It will be interesting to examine how TaHIPP3 affects spike development in the future and if the role of HIPP3 is also conserved across other crops.

Apart from regulation of spike architecture by TaAIRP2-1B, TaCol-B5 was also reported to modify spike architecture and enhance grain yield in wheat (Zhang *et al.*, 2022). Constitutive overexpression of the dominant *TaCol-B5* allele in common wheat increases not only the spike length, but also the spikelet number and tiller number, thereby improving grain yield. However, it remains unknown if and how TaAIRP2-1B affects agronomic traits other than spike length, given that *TaAIRP2-1B* is universally expressed at seedling and joint stages of wheat. Moreover, it will also be very interesting to know if and how the TaAIRP2-1B-centred network interacts with the TaCol-B5 network in the regulation of spike architecture. All in all, there is no doubt that this study is another important stepping stone towards designing future wheat with continuously increasing yield potential.
